# Self‐ and intra‐morph incompatibility and selection analysis of an inconspicuous distylous herb growing on the Tibetan plateau (*Primula tibetica*)

**DOI:** 10.1002/ece3.3151

**Published:** 2017-06-15

**Authors:** Xian‐Feng Jiang, Qing‐Jun Li

**Affiliations:** ^1^ Key Laboratory of Tropical Forest Ecology Xishuangbanna Tropical Botanical Garden Chinese Academy of Sciences Mengla China; ^2^ University of Chinese Academy of Sciences Beijing China; ^3^ Laboratory of Ecology and Evolutionary Biology State Key Laboratory in Conservation and Utilization of Bioresources in Yunnan Yunnan University Kunming Yunnan China

**Keywords:** distyly, floral display, pollinator‐mediated selection, *Primula tibetica*, self‐ and intra‐morph incompatibility

## Abstract

There is discussion over whether pollen limitation exerts selection on floral traits to increase floral display or selects for traits that promote autonomous self‐fertilization. Some studies have indicated that pollen limitation does not mediate selection on traits associated with either pollinator attraction or self‐fertilization. *Primula tibetica* is an inconspicuous cross‐fertilized plant that may suffer from pollen limitation. We conducted a selection analysis on *P. tibetica* to investigate whether pollen limitation results in selection for an increased floral display in case the evolution of autonomous self‐fertilization has been difficult for this plant. The self‐ and intra‐morph incompatibility features, the capacity for autonomous self‐fertilization, and the magnitude of pollen limitation were examined through hand‐pollination experiments. In 2016, we applied selection analysis on the flowering time, corolla width, stalk height, flower tube length, and flower number in *P. tibetica* by tagging 76 open‐pollinated plants and 37 hand‐pollinated plants in the field. Our results demonstrated that *P. tibetica* was strictly self‐ and intra‐morph incompatible. Moreover, the study population underwent severe pollen limitation during the 2016 flowering season. The selection gradients were found to be significantly positive for flowering time, flower number, and corolla width, and marginally significant for the stalk height. Pollinator‐mediated selection was found to be significant on the flower number and corolla width, and marginally significant on stalk height. Our results indicate that the increased floral display may be a vital strategy for small distylous species that have faced difficulty in evolving autonomous self‐fertilization.

## 1 INTRODUCTION

The evolution of flowers is considered to be closely associated with the pollination environment (Harder & Barrett, 2006). Flowers, the reproductive organs of angiosperms, vary significantly in morphology and are subjected to different selection pressures depending on the pollination environment. For example, wind‐pollinated plants produce light pollen that can be easily carried by wind, and the stigmas of the female wind‐pollinated flowers are exposed to the air to obtain the pollen grains more easily (Friedman & Barrett, 2009; Whitehead, 1969). Moth‐pollinated plants produce white flowers that bloom at night and generate floral scents, two features that are consistent with the foraging behaviors of moths (Boberg et al., 2014; Knudsen & Tollsten, 1993). An evolutionary transition between self‐ and cross‐fertilization may also be accompanied by the modification of floral morphological characters. Self‐fertilized plants usually produce small flower and fewer rewards, whereas cross‐fertilized plants preferentially produce big, showy flowers to attract pollinators. For instance, Vos, Wüest, and Conti (2014) demonstrated that the evolution of self‐fertilization from cross‐fertilization in *Primula* is accompanied by smaller and less conspicuous flowers.

Pollen limitation can cause severe reductions in ovule number and resources (Ashman et al., 2014; Knight et al., 2005; Totland & Sottocornola, 2001) and is the major selecting force acting on the flower through the female fitness of plants (Chapurlat et al., 2015; Sletvold, Grindeland, & Ågren, 2010; Totland, 2001). Various flower traits are demonstrated to be selected for by pollen limitation, including flower size (Sletvold et al., 2010), flower number (Parachnowitsch & Kessler, 2010), flower color (Sletvold, Trunschke, Smit, Verbeek, & Ågren, 2016), inflorescence height (Ågren, Hellström, Toräng, & Ehrlén, 2013), spur length (Chapurlat et al., 2015; Sletvold & Ågren, 2010), and flower tube length (Alexandersson & Johnson, 2001). Severe pollen limitation selects for traits that increase the floral display (Ashman & Morgan, 2004) but also selects for traits that promote autonomous self‐fertilization (Morgan & Wilson, 2005; Porcher & Lande, 2005). The directions of the selection mediated by pollen limitation on the increase in floral displays are strikingly different from those that promote autonomous self‐fertilization. This difference is because increased autonomous self‐fertilization tends to weaken self‐incompatibility, reduce herkogamy, and produce small flowers, whereas an increase in the floral display requires a big, showy corolla and a conspicuous inflorescence (Vos et al., 2014). However, a study by Fishman and Willis (2007) on selection in *Mimulus guttatus,* suggests that pollen limitation does not mediate selection on characters associated with either pollinator attraction (corolla width) or self‐fertilization (herkogamy). Thus, more case studies are required to improve our understanding of how pollen limitation mediates selection on the floral traits of plants.

Angiosperm cross‐fertilization relies on various agents, such as bees, birds, or bats, for the delivery of male gametes (pollen grains) from the anther to the stigmas of other individuals (Harder & Barrett, 2006), whereas autonomous self‐fertilization provides reproductive assurance for the flowering plants in habitats that lack pollinators (Carlson, Gisler, & Kelso, 2008; Zhang & Li, 2008). Thus, it is widely acknowledged that pollen limitation is more commonly found in flowering plants with obligate out‐crossing (Larson & Barrett, 2000). Distyly is one of the most common floral features that promote cross‐fertilization in angiosperms (Barrett & Shore, 2008). The distylous syndrome is characterized by flowers in which male and female organs are reciprocally placed between long‐ and short‐styled morphs within a population (reciprocal herkogamy); this syndrome is usually accompanied by heteromorphic self‐ and intra‐morph incompatibility (heteromorphic SI system) (Barrett & Shore, 2008; Charlesworth & Charlesworth, 1979). *Primula* is a typical distylous genus that is highly cross‐fertilized (Richards, 2003) and is evolving from cross‐fertilized distyly toward self‐fertilized homostyly (Mast, Kelso, & Conti, 2006; Vos et al., 2014). However, fewer than 10% of *Primula* species have been found to be self‐fertilized and homostylous (Richards, 2003). *Primula* is an ideal species to investigate the question of how pollinator‐mediated selection occurs on the obligate out‐crossing plant. To date, only two studies have addressed selection pressures on *Primula* (Ågren, Fortunel, & Ehrlén, 2006; Ågren et al., 2013; Li et al., 2017).

The evolution of self‐fertilization in distylous species requires the generation of a self‐fertilized homostylous variant via genetic recombination (Barrett & Shore, 2008); before this event can occur, the investment in characters that improve reproductive assurance by, for example, reducing herkogamy and weakening the SI system, would be redundant if the plant species is strictly distylous. Meanwhile, small, inconspicuous plants would probably not be attractive enough for pollinators (Grindeland & Sletvold, 2005; Peakall & Handel, 1993). Thus, it would be a good approach for the small distylous herb to increase its female fitness by increasing its floral display. We therefore raised the question of whether pollen limitation selects on the traits that increase the floral display in a case in which a small, inconspicuous plant undergoes obligate out‐crossing and faces difficulty in evolving self‐fertilization. *Primula tibetica* is an inconspicuous herb distributed in the Himalayas where lacks pollinators (Duan, Zhang, & Liu, 2007; Zhao, Du, Zhou, Wang, & Ren, 2006). Thus, this species may undergo severe pollen limitation in the field, making it an ideal model system to resolve this question. Two specific questions are addressed: (1) what is the incompatibility feature of *Primula tibetica*? and (2) how does pollinator‐mediated selection on *Primula tibetica* occur? The date at which flowering began and four morphological traits (stalk height, flower number, corolla width, and flower tube length) were included in the selection analysis, and female fitness was considered the dependent variable.

## 2 MATERIALS AND METHODS

### 2.1 Study species and site

**Figure 1:**
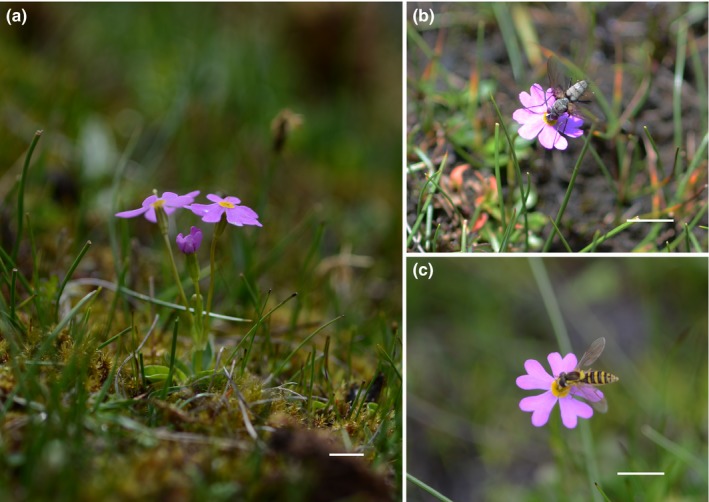
(a) Flowering plants of the long‐styled morph of *Primula tibetica*, (b) a flower of *Primula tibetica* being visited by a tachinid fly, and (c) a flower of *Primula tibetica* being visited by a syrphid fly

*Primula tibetica* is a small perennial herb belonging to sect. *Alurita* of *Primula* (Figure 1a; Richards, 2003). The stigma of the long‐styled morph rises slightly above the corolla, and the stigma of short‐styled morph is located half way down the floral tube. The anther and stigma are reciprocally placed in the long‐styled morph and the short‐styled morph. *Primula tibetica* is predominantly found in Tibet, north India, Nepal, and Bhutan along the range of the Himalayas (Ren et al., 2017; Richards, 2003). Each individual of *P. tibetica* generally produces 1–6 pink flowers. Each flower lasts for 8–12 days, and an individual plant flowers for 15–20 days. *P. tibetica* is an inconspicuous perennial herb that grows in moist meadows between 3,000 and 4,000 m.a.s.l. (Richards, 2003). Based on in‐field measurements, the average height of the stalk is only 50 ± 1.23 mm. The flowering season of *P. tibetica* lasts from early May to late June, and fruiting lasts from late May to August. According to our observations during the peak flowering season of 2016, *P. tibetica* was visited by bumble bees (*Bombus richardis*), butterflies (*Aporia goutellei*), a species of Tachinidae (Figure 1b), and a species of Syrphidae (Figure 1c). Our study site was located at Lulang town, Linzhi city, Tibet (N29°33.675′; E94°44.675′, 3,328 m.a.s.l.) near a small Tibetan village. The population of *P. tibetica* co‐occurred with other species of *Primula* (such as *P. chungensis*,* P. alpicola,* and *P. florindae*).

### 2.2 SI system and the capacity of autonomous self‐fertilization

Four treatments were included in our hand‐pollination experiment: hand self‐fertilization, autonomous self‐fertilization, hand intra‐morph pollination, and hand inter‐morph pollination. Both the long‐styled morph and short‐styled morph were subjected to the four treatments. In the early flowering season, 80 plants of each of the long‐styled and short‐styled morphs were randomly selected to be moved to a pollinator‐free house. As the flower tube of *P. tibetica* was too narrow to be manipulated, especially for the flowers of the long‐styled morph, emasculation would inevitably destroy the female organ. Thus, we were not able to emasculate the maternal flowers before hand‐pollination. However, at the end of the experiment, we discovered that neither of the two morphs of *P. tibetica* could be autonomously self‐fertilized.

On the first day that the maternal plant flowered, we picked out the anthers from the paternal flower using forceps and carefully touched the stigma of the maternal flower with the anthers. The stigmas of the long‐styled morphs were raised slightly above the mouth of the flower tube, which allowed us to immediately pollinate the flowers of the long‐styled morph. To pollinate the flowers of the short‐styled morph, we carefully withdrew the corolla of the maternal flower and touched the stigma using the anther of the paternal flower.

Mature fruits were collected 30 days after hand‐pollination, and the seeds of each capsule were counted. The results of the hand‐pollination experiments were so evident that we did not use complicated statistical methods. The capacity for autonomous self‐fertilization in each morph of *P. tibetica* was calculated by dividing the average seed production following inter‐morph pollinations by the average seed production following autonomous self‐fertilizations. The index of self‐incompatibility proposed by Lloyd and Schoen (1992) and further modified by Ferrero *et al*., (2011) was applied to infer the degree of the heteromorphic SI system of *P. tibetica* for both the short‐styled and long‐styled morphs independently. The self‐incompatibility index (SCI) was calculated as follows: The average seed production per capsule following hand inter‐morph pollinations was divided by the average seed production per capsule following hand self‐pollinations. The intra‐morph incompatibility index (MCI) was calculated as follows: The average seed production per capsule of hand intra‐morph pollination was divided by the average seed production of the hand inter‐morph pollination.

### 2.3 Phenotypic selection on *P. tibetica*

**Figure 2:**
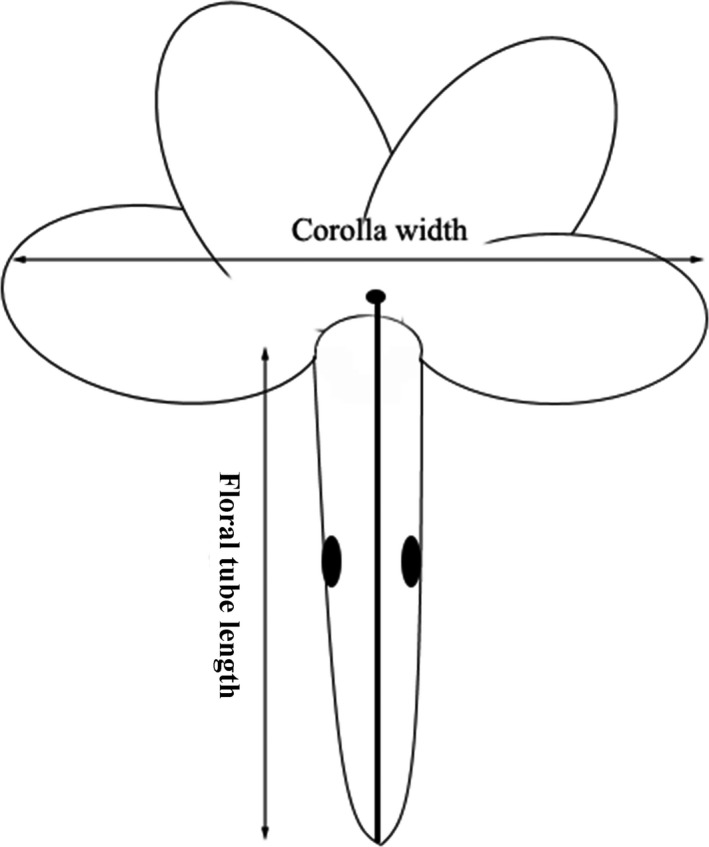
A sketch plot showing the measured corolla width and the floral tube length of *Primula tibetica*. The corolla width was measured across the widest part of the corolla, and the floral tube length was determined from the base to the mouth of the tube

From 15 May to 28 June 2016, five plants of each of the long‐styled and short‐styled morphs of *P. tibetica* were marked daily by sticking a numbered plastic tag on the ground near the rosette of the plant in the field. Because hand‐pollination of the short‐styled morph would inevitably destroy the flower tube, two different tagged individuals of the long‐styled morph were hand‐pollinated daily to measure the selective force exerted by the pollinators and the magnitude of pollen limitation on *P. tibetica*. We marked the date of the start of flowering on the tag of each individual to understand the effects of phenology on *P. tibetica*. The resulting data were transformed into the number of days from 1 January 2016, in the subsequent statistical analysis. Meanwhile, four morphological traits (the number of flowers, corolla width, stalk height, and floral tube length) were measured to understand the selection pressure on the floral morphology of *P. tibetica*. The corolla width and flower tube length were determined by measuring 1–3 fully open flowers on each tagged individual with the aid of digital calipers following the criteria outlined in Figure 2. The mature infructescence of each tagged individual was collected from the basal rosette at 30th July, the number of flowers of each inflorescence was counted based on the stalks persisting on each infructescence, and the seeds in each capsule were counted. The height of the stalk was determined by measuring the lengths of the stalks that persisted on the infructescence with a ruler.

A large number of plants were destroyed by domesticated mammals. In total, 76 open‐pollinated plants (36 plants of the long‐styled morph and 40 plants of the short‐styled morph) and 39 hand‐pollinated plants were collected and included in the phenotypic selection analysis. The magnitude of pollen limitation was calculated as follows: 1—the average seed production per capsule of hand‐pollinated plants divided by the average seed production per capsule of open‐pollinated plants.

*T*‐tests were used to infer the statistical significance of the differences in flowering time, flower number, stalk height, floral tube length, corolla width, average seed production per capsule, and total seed production per individual between open‐pollinated plants and hand‐pollinated plants. The correlations between the measured traits were determined with Pearson's correlation analysis. The total number of seeds per individual was used to assess the female fitness of a plant. The selection analysis was conducted following a method by Lande and Arnold (1983) in which relative fitness was used as the response variable and standardized trait values (mean value = 0, variance = 1) were used as the explanatory variables separately for open‐pollinated plants and hand‐pollinated plants. First, we applied a full quadratic model to the five measured traits of the open pollinated plants, but no correlational or quadratic selection pressure was found. Therefore, a linear model was applied for the selection analysis. The variance inflation factor (VIF) of each trait in the linear model was checked to assess the degree of multicollinearity, and the VIFs of all variants in the linear model were determined to be less than 3.5; this result indicated that the multicollinearity was not severe in our model (the threshold is generally 10). To assess the strength of the selective pressure exerted by the pollinators, we determined the pollinator‐mediated selection gradient (β_poll_) by subtracting the linear selective gradient of hand‐pollination (β_HP_) from the linear net selective gradient (β_n_), that is, β_poll_ = β_n_–β_HP_. The statistical significance of the difference between the selective gradient of the hand‐pollinated plants (β_HP_) and that of the open‐pollinated plants (βn) was tested with an ANCOVA. We did not apply the full quadratic model to the hand‐pollinated plants because the sample size was quite limited. Selection gradients were illustrated with added‐variable plots, in which the residual from a linear regression model of relative fitness on all traits except the focal trait was plotted against the residuals from a regression model of the focal trait on the other traits. All statistical analyses were conducted in SAS 9.2, and the data are shown as the Means ± *SE*.

## 3 RESULTS

### 3.1 SI system and the capacity of autonomous self‐fertilization

**Figure 3:**
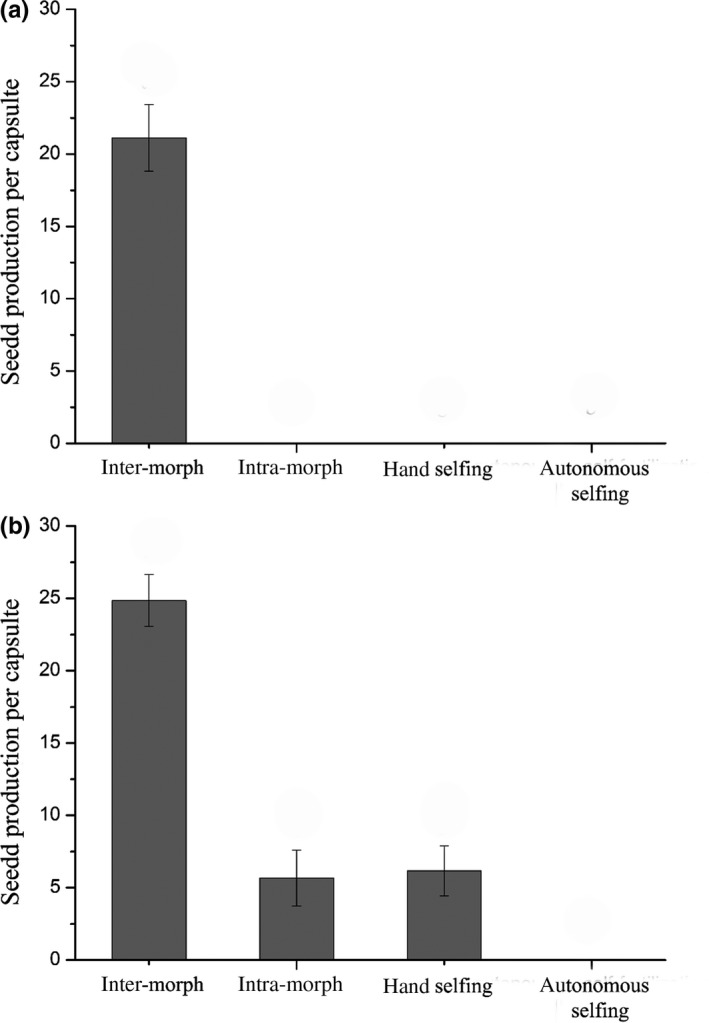
The mean seed productions following the hand‐pollination treatments in *Primula tibetica* are plotted. (a) The mean seed production following the hand‐pollination treatment with the short‐styled morph as the maternal parent (inter‐morph pollination, 21.12 ± 2.30; intra‐morph pollination, 0; intra‐flower pollination, 0; autonomous self‐fertilization, 0). (b) The mean seed production following the hand‐pollination treatment with the long‐styled morph as the maternal parent (inter‐morph pollination, 24.85 ± 1.80; intra‐morph pollination, 5.67 ± 1.93; intra‐flower pollination, 6.16 ± 1.74; autonomous self‐fertilization, 0)

The differences among pollination treatments were evident, and two of the four levels for the short‐styled morph showed zero values (hand inter‐morph mating, 21.13 ± 2.29; hand intra‐morph mating, 0; hand selfing, 0; and autonomous self‐fertilization, 0). For the long‐styled morph, one treatment produced zero seeds, and the differences among the remaining treatments were sharply contrasted (hand inter‐morph mating, 24.85 ± 1.80; hand intra‐morph mating, 5.67 ± 1.93; hand selfing, 6.16 ± 1.74; and autonomous self‐fertilization, 0) (Figure 3). The SCI and MCI values of the short‐styled morph were 0, whereas the SCI and MCI values of the long‐styled morph were 0.25 and 0.23, respectively. The capacity of autonomous self‐fertilization in both the long‐ and short‐styled morphs of *P. tibetica* was 0.

### 3.2 Phenotypic selection on *P. tibetica*

**Figure 4:**
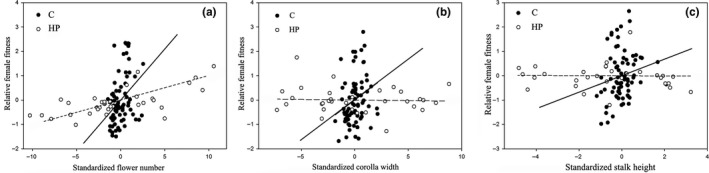
Standardized linear phenotypic selection gradients for (a) the flower number, (b) corolla width, and (c) stalk height of *Primula tibetica* in open‐pollinated plants (C; filled symbols, solid line) and in hand‐pollinated plants (HP; open symbols, dashed line) during the 2016 flowering season. The selection gradient is illustrated as an added‐variable plot in which the residuals from a linear regression model of the relative fitness of all traits except the focal trait are plotted against the residuals from a regression model of the focal trait on the other traits

In the 2016 flowering season, the magnitude of pollen limitation of *P. tibetica* was 0.32 in the studied population. Stalk height was significantly positively correlated with other traits (Table 1). The total seed production per individual and the mean seed production per capsule were significantly different between open‐pollinated plants and hand‐pollinated plants. No significant differences were found in terms of flowering time or the four measured morphological traits between open‐pollinated plants and hand‐pollinated plants (flower number, stalk height, flower tube length, and corolla width) (Table 2). We found significant positive selection gradients for the flowering time (*p *=* *.00157), flower number (*p *=* *.0014), and corolla width (*p *=* *.021) (Table 3). The selection pressure on the stalk height was found to be marginally significant (*p *=* *.099) (Table 3). The difference of the selection gradients between open‐pollinated plants and hand‐pollinated plants was statistically significant for the corolla width (*p *<* *.001) and flower number (*p *=* *.002) (Table 3, Figure 4a,b). The pollinator‐mediated selection on the stalk height was marginally significant (*p *=* *.098, Table 3 and Figure 4c).

**Table 1:** Pearson's correlation analysis was applied to analyze the correlations between the five traits (flowering start, flower number, corolla width, stalk height and the floral tube length) in *Primula tibetica*. Data were pooled from both open‐pollinated plants and hand‐pollinated plants

	No. of flower	Corolla width	Floral tube length	Stalk height
Flowering start	0.11	0.15	0.15	0.68***
No. of flower		0.32***	0.24*	0.25**
Corolla width			0.31***	0.44***
Floral tube length				0.26**

*.01 < *p *<* *.05, **.01 < *p *<* *.0001, ***p <* *.0001.

**Table 2:** Comparison of the five traits (corolla width, flower number, flowering start, floral tube length, and stalk height) and the average seed production per capsule of the open‐pollinated plants and hand‐pollinated plants using *t*‐tests. The data are shown as the Means ± *SE*. Statistically significant traits are marked in bold

	Open‐pollinated plants (*n*)	Hand‐pollinated plants (*n*)	*T* value	*p*
Corolla width (mm)	10.76 ± 0.15 (76)	10.46 ± 0.18 (39)	1.20	.23
Flower number	2.34 ± 0.13 (76)	2.38 ± 0.16 (39)	−0.17	.87
Flowering start (No. of days from 2016/1/1)	154.43 ± 1.48 (76)	159.97 ± 2.21 (39)	1.79	.07
Flower tube length (mm)	5.67 ± 0.07 (76)	5.52 ± 0.07 (39)	1.33	.19
Stalk height (mm)	43.17 ± 2.95 (76)	42.24 ± 3.23 (39)	0.19	.85
Mean seed production per capsule	9.79 ± 1.14 (76)	14.36 ± 1.13 (39)	−**2.84**	**.006**
Total seed production per individual	21.51 ± 2.82 (76)	35.42 ± 4.84 (39)	**2.63**	**.0098**

**Table 3:** The direction selective gradients (β + *SD*) on the flowering time, flower number, corolla width and floral tube length in the open‐pollinated and the hand‐pollinated *P. tibetica* plants, as implemented in the linear multiple regression model. The differences between the selective gradients on the natural plants and on the hand‐pollinated plants were tested by ANCOVA

	Open‐pollinated plant (*n*)	Hand‐pollinated plant (*n*)	Pollinated mediated selection	*p*
	β_n_	β_HP_	β_poll_	
Flowering time	−**0.43 ± 0.17** * (76)	−0.01 ± 0.02 (39)	−0.42	.637
Flower number	**0.42 ± 0.13** ** (76)	**0.10 ± 0.02** *** (39)	**0.41**	**<.001**
Corolla width	**0.33 ± 0.14** * (76)	−0.005 ± 0.02 (39)	**0.335**	**.002**
Floral tube length	−0.04 ± 0.12 (76)	0.03 ± 0.02 (39)	−0.07	.352
Stalk height	0.34 ± 0.21 (76)	0.003 ± 0.02 (39)	0.337	.098

*p <* *.05, **p <* *.01, and ***p <* *.0001. Statistically significant traits are marked in bold.

## 4 DISCUSSION

*Primula tibetica* was demonstrated to be highly self‐ and intra‐morph incompatible and was unable to autonomously self‐fertilize (Figure 3). These results indicate that *P. tibetica* highly relies on pollinators for sexual reproduction. We demonstrated that *P. tibetica* underwent severe pollen limitation during the 2016 flowering season. Our evidence suggested that the traits correlated with the floral display (flower number, corolla width, and stalk height) were positively selected by pollinators in the population of 2016 (Table 3 and Figure 4). Thus, we can reasonably conclude that *P. tibetica* was under selective pressure to increase its floral display.

### 4.1 SI system and autonomous self‐fertilization

We demonstrated that *P. tibetica* was highly self‐ and intra‐morph incompatible. We found that the heteromorphic SI system of *P. tibetica* was extremely strong, and neither the long‐styled nor the short‐styled morph of *P. tibetica* was capable of autonomous self‐fertilization. In many primrose species, the heteromorphic SI system is stronger in the short‐styled morph than in the long‐styled morph (Ernst, 1955), which may be due to the genetic background of the distylous syndrome (Barrett & Shore, 2008; Charlesworth & Charlesworth, 1979). Although the self‐ and intra‐morph pollinated long‐styled morphs could produce a small number of seeds, the capacity for autonomous self‐fertilization in the long‐styled morph was zero (Figure 3b). This result indicated that the autonomous self‐fertilization was not only limited by the heteromorphic SI system but also strongly limited by the reciprocal herkogamy. As the self‐compatible homostylous variant has been demonstrated to occur only through genetic recombination instead of being gradually selected by pollinators (Barrett & Shore, 2008), we suggest that *P. tibetica* will face difficulty in evolving self‐fertilization by lessening the degree of herkogamy and weakening the magnitude of the SI system under the pressure of pollen limitation.

### 4.2 Selection on flowering time and flower tube length

Flowering time undergoes selection due to temporal variations in pollinator and herbivore abundances (Chapurlat et al., 2015). The abundance of pollinators is generally low in early spring; thus, the flowers that open in early spring may undergo greater pollen limitation than those that open during summer (Dupont, Padrón, Olesen, & Petanidou, 2009). Our results suggest that the selection gradient for the flowering time of *P. tibetica*, although significant, was not significantly mediated by pollinators (Table 3). Indeed, few pollinators visited *P. tibetica* throughout the flowering season (Pers. Obs. 2016). This is likely to be because *P. tibetica* was less attractive compared with other plants flowering synchronously in the same habitat (including *Primula florinda*,* Iris tibetica,* and several species of *Pedicularis*). Furthermore, our unpublished data demonstrate that *P. florinda* was less pollen‐limited than *P. tibetica* over the same period in 2016. The study population of *P. tibetica* was also severely damaged by the trampling or grazing of domesticated mammals (horses, yaks, and pigs) in 2016; thus, we assumed that the negative selection gradient for the flowering time of *P. tibetica* may be mediated by animal grazing or trampling.

The length of the flower tube is highly correlated with the proboscis length of pollinators and thus is related to pollination efficiency. We did not find significant selection pressure on the flower tube length in *P. tibetica*. According to our observations during the 2016 flowering season, *P. tibetica* was visited by both long‐tongued pollinators, such as butterflies and bumble bees, and short‐tongued pollinators, such as flies and syrphid flies. The most frequently found pollinator of *P. tibetica* was a pollen‐feeding Tachinidae fly (Figure 1b). Thus, the variation in the length of the flower tube may not greatly limit the visits of pollinators.

### 4.3 Selection on traits involved in the floral display

Maximizing the floral display increases the attractiveness of flowering plants to pollinators and is especially important for obligate out‐crossing species. A variety of morphological traits, including the flower number, corolla size, and plant height, are related to the floral display (Chapurlat et al., 2015; Sletvold et al., 2010). We found significantly positive selection gradients for the corolla width and flower number of *P. tibetica* as well as marginally significant selection pressure on the stalk height (Table 3 and Figure 4). The hand‐pollinated treatment did not change the morphological traits of *P. tibetica* significantly (Table 2). The selection gradients for the flower number and the corolla width were significantly different between open‐pollinated and hand‐pollinated plants. Meanwhile, the pollinators contributed 97.6% to the net selection gradient for the flower number and explain the entire natural selection gradient for the corolla width (Table 3); these results indicate that the selection pressures on the flower number and corolla width of *P. tibetica* were mediated by the pollinators (Table 3, Figure 4a,b). The pollinator‐meditated selection on the stalk height was marginally significant, and pollinators contributed to 99.12% of the net selection gradient for the stalk height of *P. tibetica* (Table 3 and Figure 4c). Thus, we assumed that the selection pressure on the stalk height of *P. tibetica* was probably pollinator mediated. The selection pressure on the stalk height was marginally significant, which likely resulted from an insufficient sample size. In conclusion, our results suggest that in the investigated population, the traits involved in the floral display were positively selected by the pollinators during the 2016 flowering season.

The direction and strength of the pollinator‐mediated selection may closely correlate with the pollination characters of the plant species, such as mating patterns and pollination environment. Various studies have examined pollinator‐mediated selection on flowering plants, and the strength and direction of the selection on the traits involved in the floral display varies between species, years, and populations. Sletvold et al. (2010) provided evidence that pollinators mediate positive selection on the plant height, corolla size, and flower number of *Dactylorhiza lapponica* even though the strength of the pollinator‐mediated selection on these traits varies between years and populations. A study of *Gymnadenia conopsea* by Chapurlat et al. (2015) indicated that pollinators mostly exert significantly positive selection on the traits involved in the floral display (flower number, plant height, and corolla size), but the selection gradient varied among populations. In that study, there was a population in which the pollinators exerted negative selection on the corolla size. Parachnowitsch & Kessler (2010) suggested that significantly positive pollinator‐mediated selection is found on the flower size and flower number of *Penstemon digitalis*, but negative pollinator‐mediated selection is found to act on the plant height (though without significance).

We found that the traits involved in the floral display (corolla width, flower number, and stalk height) were selected positively by the pollinators. Our results indicated that the increase of the floral display would be especially important for *P. tibetica*, an inconspicuous out‐crossing flowering species that grows in the Tibetan plateau. We assumed it might be because the evolution of self‐fertilization for *P. tibetica* would need to reduce the herkogamy and weaken the dimorphic SI system at the same time, and these two traits may be not significantly selected by the pollinators for the distylous species. Therefore, there would be redundancy in the investment on the traits for reproductive assurance (such as reducing the herkogamy or weakening the heteromorphic SI system) as *P. tibetica* is difficult evolving toward self‐fertilization. Meanwhile, because *P. tibetica* is inconspicuous compared with the co‐occurring species, an increase in the floral display would greatly promote pollinator attraction. Consequently, for *P. tibetica,* increase in the floral display to attract pollinators would be a better strategy than selection on the traits correlated with reproductive assurance.

## 5 CONCLUSION

During the 2016 flowering season, we found significantly positive selection gradients for traits involved in the floral display (corolla width, flower number, and stalk height) in the focal population (Table 3 and Figure 4). Although the sample size of hand‐pollinated plants was relatively low, we could reasonably conclude that the traits involved in the floral display of *P. tibetica* were positively selected by pollinators. Our results suggested that enlarging the flower display may be vital for this inconspicuous distylous plant. This study, as one of few to analyze selection on this small, distylous herb that grows in the Tibetan plateau, provides evidence that traits involved in the floral display are especially important for such small cross‐fertilized alpine plants.

## CONFLICT OF INTEREST

None declared.
